# Voluntary Exercise Ameliorates Chronic Ethanol Withdrawal-Induced Adaptations of Opioid Receptor Expression in the Nucleus Accumbens, Dopamine Release, and Ethanol Consumption

**DOI:** 10.3390/biomedicines12071593

**Published:** 2024-07-17

**Authors:** Christina A. Nelson, James N. Brundage, Benjamin M. Williams, Jared K. Baldridge, Alyssa L. Stockard, Charlton H. Bassett, Brandon J. Burger, Bridger T. Gunter, Andrew J. Payne, Jordan T. Yorgason, Scott C. Steffensen, Kyle B. Bills

**Affiliations:** 1 Department of Biomedical Sciences, Noorda College of Osteopathic Medicine, Provo, UT 84606, USA; casmall@noordacom.org (C.A.N.); kbbills@noordacom.org (K.B.B.); 2 Department of Psychology/Neuroscience, Brigham Young University, Provo, UT 84602, USAjared.baldridge1@gmail.com (J.K.B.); alstockard24@gmail.com (A.L.S.);

**Keywords:** alcohol-use disorder (AUD), voluntary exercise, kappa opioid receptors (KORs), delta opioid receptors (DORs), nucleus accumbens (NAc), ventral tegmental area (VTA), exercise therapy, opiate receptor adaptation

## Abstract

Exercise has increasingly been recognized as an adjunctive therapy for alcohol-use disorder (AUD), yet our understanding of its underlying neurological mechanisms remains limited. This knowledge gap impedes the development of evidence-based exercise guidelines for AUD treatment. Chronic ethanol (EtOH) exposure has been shown to upregulate and sensitize kappa opioid receptors (KORs) in the nucleus accumbens (NAc), which is innervated by dopamine (DA) neurons in the midbrain ventral tegmental area (VTA), which may contribute to AUD-related behaviors. In this study, we investigated the impact of voluntary exercise in EtOH-dependent mice on EtOH consumption, KOR and delta opioid receptor (DOR) expression in the NAc and VTA, and functional effects on EtOH-induced alterations in DA release in the NAc. Our findings reveal that voluntary exercise reduces EtOH consumption, reduces KOR and enhances DOR expression in the NAc, and modifies EtOH-induced adaptations in DA release, suggesting a competitive interaction between exercise-induced and EtOH-induced alterations in KOR expression. We also found changes to DOR expression in the NAc and VTA with voluntary exercise but no significant changes to DA release. These findings elucidate the complex interplay of AUD-related neurobiological processes, highlighting the potential for exercise as a therapeutic intervention for AUD.

## 1. Introduction

### 1.1. Significance

Alcohol use disorder (AUD) is a chronically relapsing disorder characterized by compulsion to seek and take alcohol and the emergence of a negative emotional valence when access to the drug is restricted. Research on alcohol use disorder (AUD) focuses on identifying the neural substrates that are highly responsive to the drug, undergo adaptation with chronic use, and contribute to ongoing drug-seeking behaviors. AUD and opioid use disorder (OUD) share similar neurobiological substrates in the brain reward system, in particular opioid receptors (ORs). In fact, the μ-OR (MOR) antagonist naltrexone (e.g., common brands: Revia and Vivitrol) used to treat OUD is one of only three FDA-approved pharmacological treatments for AUD.

### 1.2. Alcohol Use Disorder and the Involvement of the Mesocorticolimbic Dopamine System

The development of AUD involves a complex interplay of genetic predispositions, environmental factors, and neurobiological factors that contribute to the persistent cycle of alcohol-seeking behavior. A leading theory posits that neurobiological basis of the addiction cycle starts with ‘binge/intoxication’ through drug reinforcing and rewarding properties [1]. The mesocorticolimbic dopamine (DA) reward system, originating in the midbrain ventral tegmental area (VTA) and projecting to the nucleus accumbens (NAc), constitute brain substrates underlying the hedonic and reinforcing effects of drugs of abuse, including alcohol. Dopamine is the canonical neurotransmitter implicated in motivated behavior and reward learning [2]. Dopamine is critical for natural behaviors such as feeding [3,4,5], drinking [6,7], and has been implicated in the habit-forming actions of several addictive drugs [8,9,10,11]. Within the mesocorticolimbic pathway, opioid receptors (ORs) exert regulatory control over release of DA, further influencing stress responses, reward processes, and related learning.

### 1.3. Opioid Receptors

Opioid receptors (ORs) are expressed both directly on VTA GABA neurons, local striatal γ-aminobutyric acid (GABA) neurons, and cholinergic interneurons (CINs) in the striatum, making them a strong potential target for modulating DA transmission in the striatum [12]. There are three traditionally recognized ORs: ΜORs, δ-ORs (DORs), and κ-ORs (KORs). Each receptor is an inhibitory G-coupled protein receptor whose activation leads to subsequent inhibition of firing rate and neurotransmission via inhibition of adenylate cyclase, reduction of calcium currents, and activation of inwardly rectifying potassium channels [13,14,15,16,17]. Kappa opioid receptors are expressed extensively in the NAc, both in the core and the shell [13,14,15], where their activation inhibits DA synthesis, release and DA neuron excitability [13,18]. Data suggests that KORs are synthesized in the VTA DA cell bodies, where they are expressed and subsequently transported to terminals in the NAc, where they are then integrated into the presynaptic membrane [16]. Activation of KORs is associated with dysphoria and stress, many argue that dysregulation of the dynorphin/KOR system contribute to the negative reinforcement of alcohol [17,19,20,21]. Evidence suggests an increased expression and activity of KORs in EtOH-dependent animals, potentially contributing to the increased seeking behavior associated with alcohol dependency [22,23]. This is demonstrated by a reduction in dependent-state seeking behavior upon administration of the KOR antagonist norbinaltorphimine (nor-BNI), with negligible impact on non-dependent animals [18]. Similarly, Anderson et al. (2018) found that in a drinking-in-the-dark (DID) model of binge drinking, administration of nor-BNI decreased EtOH preference and blood EtOH content (BEC) while administration of the KOR agonist U-50488 increased EtOH preference and BEC [24]. They also indicate that decreased EtOH preference is observed when nor-BNI or a DYN/KOR inhibitory DREADD viral constructs were injected directly into the central amygdala [24]. Additionally, Chefer et al. (2013) indicated that this KOR-mediated aversive behavior is VTA DA neuron dependent [25]. Such findings indicate involvement of KORs in the synaptic adaptations that are characteristic of dependency [17]. It is important to note that dysregulation of the DYN/KOR system and resulting dysregulation of DA signaling has been found in adult humans with AUD [26] as well as changes to endogenous opioid release in response to alcohol consumption in heavy drinkers [27].

In contrast, CINs, and GABAergic medium spiny neurons (MSNs) in the NAc express MORs and DORs [28,29,30,31]. In addition, VTA GABA neurons are generally involved in the inhibition of VTA DA neurons, wherein activation of MORs and DORs leads to disinhibition of DA neurons and a subsequent increase in DA release in the NAc [32,33,34]. At the behavioral level, activation of MORs and DORs produce feelings of euphoria and problems with coordination [35], while KORs produce dysphoria [36]. Therefore, it is suggested that an upregulation of KORs and downregulation of MORs and DORs with alcohol exposure results in the behavior changes that lead to dependence.

### 1.4. Exercise

Compelling evidence highlights the advantageous role of aerobic exercise as an adjunct to existing pharmacological and psychological treatment protocols for AUD [37]. Exercise elicits a cascade of physiological changes relevant to the mesolimbic system. Similar to the acute effects of EtOH consumption, aerobic exercise has been observed to increase levels of tyrosine hydroxylase, the rate limiting enzyme in DA synthesis in the NAc [38,39]. While prevailing evidence predominantly advocates for the ameliorative role of exercise [40,41], certain studies suggest that excessive exercise may exacerbate addictive tendences [22,42]. Furthermore, 6-weeks of voluntary wheel running in rats increased D2 auto-receptor density in the NAc [39]; a modification associated with increased vulnerability to addictive behavior [43,44,45]. Currently, these discrepancies are not well understood; however, disparate recommendations might be to blame due to the inconsistencies in methodology, type, intensity, and duration of treatment protocols. Evidence supporting opioid receptor changes with exercise is less conclusive and appears to vary significantly by region [46], notably lacking in the mesolimbic system [19,47]. To date, direct measurement of KOR and DOR alterations in the ventral striatum in the context of exercise and EtOH remains unexplored. In this study, we investigated the effect of chronic exercise on KOR and DOR sensitivity and expression and hypothesized that voluntary wheel-running will down-regulate KORs and downregulate DORs in the NAc. Furthermore, we hypothesized that exercise would prevent EtOH-induced sensitization of KORs and reduce EtOH consumption in chronic EtOH-exposed mice.

## 2. Material and Methods

### 2.1. Animals

Male C57BL/6J (Jackson Labs, Bar Harbor, ME, USA; aged 6–12 weeks) were given ad libitum access to food and water and were maintained on a reverse 12:12-h light/dark cycle (lights on at 15:00 h). Mice were randomly assigned to one of four cohorts: EtOH without exercise, EtOH with exercise, saline with exercise, and saline without exercise. Mice were injected twice daily with EtOH (2.0 g/kg; 16% *w*/*v*; IP) or an equivalent volume of saline for 14 days. Exercise groups were given ad libitum access to a running wheel, whereas the exercise wheels in the non-exercise control groups had zip ties restricting wheel rotation to prevent exercise. Intoxication was visually verified with loss of consciousness, which decreased visually with dependence [48]. All protocols and animal care procedures were in accordance with the National Institutes of Health Guide for the Care and Use of Laboratory Animals and approved by the Brigham Young University Institutional Animal Care and Use Committee. All efforts were made to minimize animal suffering and the number of animals used in the present study.

### 2.2. Brain Slice Preparation

Isoflurane 4% (Patterson Veterinary, Devens, MA, USA) anesthetized mice were sacrificed by decapitation and brains were rapidly removed and transferred into ice-cold, pre-oxygenated (95% O_2_/5% CO_2_) artificial cerebral spinal fluid (aCSF), consisting of (in mM): NaCl (126), KCl (2.5), NaH2PO4 (1.2), CaCl2 (2.4), MgCl2 (1.2), NaHCO3 (25), glucose (11), with pH adjusted to 7.4. Tissue was sectioned into 220 μm-thick coronal slices containing the striatum with a vibrating tissue slicer (Leica VT1000S, Vashaw Scientific, Norcross, GA, USA). Brain slices were placed in a submersion recording chamber and perfused at 1 mL/min at 36 °C with oxygenated aCSF.

### 2.3. Fast Scan Cyclic Voltammetry

Fast scan cyclic voltammetry (FSCV) recordings of DA signals were performed and analyzed using Demon Voltammetry (Wake Forest, NC, USA, version 2024) and LabView Software (version 2.2) (National Instruments, Austin, TX, USA) [49]. The carbon fiber electrode (7 μm × ~150 μm) potential was linearly scanned as a triangular waveform from −0.4 to 1.2 V and back to −0.4 V (Ag vs. Ag/Cl) at a scan rate of 400 V/s. Cyclic voltammograms were recorded at the carbon fiber electrode every 100 ms by means of a potentiostat (Dagan Corporation, Minneapolis, MN, USA). Dopamine release was evoked every 2 min through a bipolar stimulating electrode. To assess baseline DA signals across increasing current simulations in input/output experiments, we collected single pulse baseline DA signals (0.5 ms, 350 μA) until stability was achieved across 4–5 collections. KORs: Baseline single pulse responses were measured, followed by bath application of the selective KOR agonist U-50,488 at 0.3 μM or 1 μM, followed by a reversal dose of 1 μM nor-BNI, a selective KOR antagonist. DORs: Baseline single pulse responses were measured, followed by bath application of the selective DOR agonist DPDPE at 1 μM.

### 2.4. Preparation of Brain Slices for Imaging and Confocal Microscopy

Mice were anesthetized using isoflurane (4%) and underwent transcardial perfusion with 4% paraformaldehyde [50]. Once perfused, brains were carefully removed and placed in 4% PFA for 24 h to facilitate continued fixation. After incubation in PFA, brains were placed in a solution of 30% sucrose in 1× PBS until the density of the brain matched that of the solution and the brains dropped to the bottom of the vial (~24–48 h). Brains were then flash frozen in dry ice and mounted on a cold microtome stage. Targeting the VTA and NAc, brains were sliced at 30 µm on the microtome, and slices were placed in cryoprotectant (30% ethylene glycol, 30% sucrose, and 0.00002% sodium azide in 0.1 M PBS) and kept at −20 °C until staining. Slices were washed three times in 1× PBS for 10 min on a rotator. They were then blocked with a blocking buffer comprised of 4% normal goat serum (Cell Signaling Technology, Danvers, MA, USA), 0.1% Triton-X 100, and 1× PBS. Slices were then washed another three times in 0.2% PBS in a rotator. Primary antibodies were applied and allowed to incubate for 20 h. Following staining, the slices were washed three times in 0.2% PBST, and secondary antibodies were applied. After a 2-h incubation period, they were washed another 3 times with 0.2% PBST and once with 1× PBS. Antibodies included mouse anti-tyrosine hydroxylase (Novus, Centennial, CO, USA, 1:1328), rabbit anti-KOR (LifeSpan Biosciences, Lynnwood, WA, USA, 1:200) and rabbit anti OPDR1 (Lifespan Biosciences, 1:100), as well as secondaries Alexa Fluor 405 donkey anti-sheep (1:900, Abcam, Cambridge, UK) and Alexa Fluor 594 goat anti-rabbit (1:500, Invitrogen, Waltham, MA, USA). To mount slides, sections were placed on microscope slides and dried for ~5 min. Once dried, a drop of vectashield (Vector Laboratories, Newark, CA, USA) was placed on the tissue, and a cover slip was placed on the slide. Slides were set overnight, and then they were kept at 4 °C until imaging. An Olympus FluoView FV1000 confocal microscope (Tokyo, Japan) was used to image mount slices. Brain slices were mounted on microscope slides and imaged under oil immersion at 40×. To ensure consistent readings between samples, a constant photomultiplier tube voltage and gain were set between all acquired images.

### 2.5. 24-Hour Two Bottle Choice Procedure

Mice were given ad libitum access to two bottles containing water over the course of one week for habituation. One bottle was randomly chosen to replace its contents with 16% EtOH/water. Bottle weights were measured several times daily for one week to determine baseline drinking behavior. To ensure that fluid volumes consumed by the mice were accurately measured, another bottle was filled with saline to access any fluid lost to gravity or from evaporation. This amount was then subtracted from the total amount of fluid drank by the mice. Following this, a 2-week period of twice daily EtOH injections (2 g/kg) was used to establish EtOH dependence. Drinking was again measured over the course of another week to determine changes to drinking behavior in conjunction with EtOH dependence.

### 2.6. Statistical Analysis

For FSCV, evoked recordings were normalized to the established baseline within subjects and then averaged across subjects in corresponding time intervals. Comparisons were made using a Dunnett’s analysis. All statistical tests were performed in Microsoft Excel (Redmond, WA, USA, version 16.86) using XLMiner Analysis ToolPak (Frontline Systems, Incline Village, NV, USA). Figures were compiled using IGOR Pro Software (Wavemetrics, Lake Oswego, OR, USA, version 8.1).

For brain slice imaging, images were loaded into NIH image software (Bethesda, MA, USA, Image J version 2.39) Images were duplicated to preserve the original settings, while color thresholding and brightness contrast adjustments were made to determine the location of cells and create regions of interest (ROIs). ROIs were then projected back onto the unedited images, where area and mean intensity were recorded for each channel. This process was performed by three independent raters who were blinded to the hypothesis. The relative density of KORs and DORs was determined by calculating the ratio of mean fluorescence intensity to area.

Two bottle choice (TBC) was analyzed measuring the daily fluid lost from each of the bottles as a measure of drinking (adjusted by a drip/evaporation control). Amounts of water/EtOH consumption was converted to g/kg based on animal’s average weight. Results were measured as the percent of EtOH consumption compared to total water consumption. Multiple comparisons ANOVA was performed in Excel using XLMiner Analysis ToolPak (Frontline Systems, Incline Village, NV, USA).

## 3. Results

### 3.1. Effects of Exercise on EtOH Two Bottle Choice Behavior in EtOH-Dependent Mice

**Figure 1:**
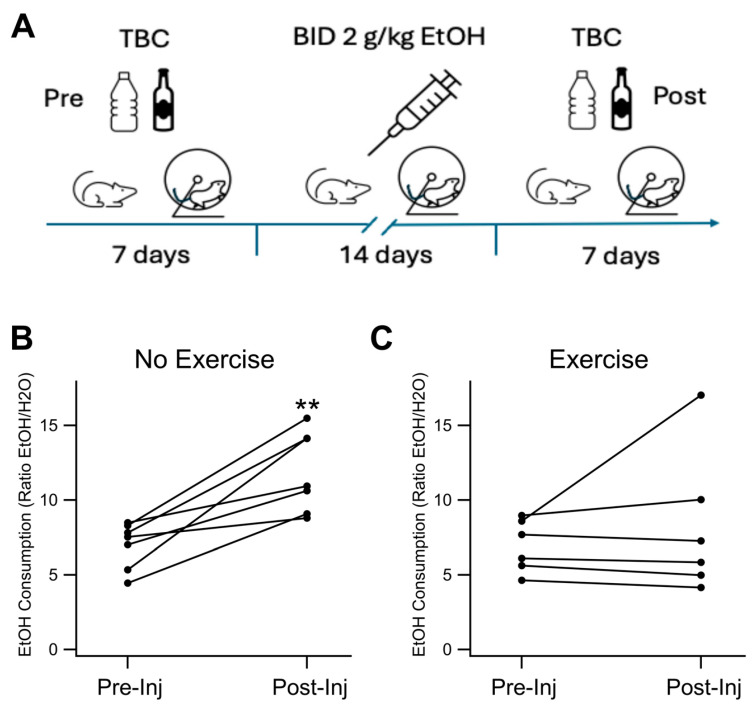
24 h 2 bottle choice. Exercise Prevents Increased EtOH Consumption in EtOH-dependent Mice: (**A**) Illustration showing two bottle choice (TBC) drinking before and after two weeks of twice-daily injections of EtOH. (**B**) Chronic EtOH injections increased EtOH consumption in the TBC procedure. (**C**) Exercise reduced EtOH consumption increase in the TBC procedure post chronic EtOH injections, measured as the percent change in ratio of consumption of EtOH:water after mice had received chronic EtOH for two weeks with or without exercise. Asterisks ** indicates *p* < 0.01.

To assess exercise and EtOH effects on drinking behavior, drinking was measured using a two-bottle choice (TBC) paradigm where mice received twice-daily EtOH injections of 2.0 g/kg daily for two weeks (Figure 1A), Running wheels were available in the mouse cages in the Exercise (EX) group (*n* = 6), but in the no exercise group (NX) (*n* = 7) the running wheels were zip-tied to prevent movement. Mice were allowed to run voluntarily in the EX group. Ethanol consumption was evaluated before injections and after as an index of dependence. We compared their % EtOH pre/post chronic EtOH. The animals that did not exercise consumed 76.1 ± 19.0% more EtOH than at baseline. Animals that exercised consumed 13.0 ± 17.4% more EtOH than their baseline. There was a significant difference between pre and post %EtOH consumption in the NX group, but not in the EX group (*F_(_*_1,12_) = 17.60, *p* = 0.0012, Figure 1B,C) There was no significant difference between the % EtOH consumption between EX and NX groups post EtOH conditioning (*F*_(1,12)_ = 2.09, *p* = 0.108, Figure 1B,C) but there was a significant difference in the % change of these % EtOH values after chronic EtOH between groups (*F*_(1,11)_ = 5.86, *p* = 0.034, Figure 1B,C). The EX mice consumed 18.5 ± 2.8 g/kg/day of EtOH and NX mice consumed 20.5 ± 4.0 g/kg/day of EtOH.

### 3.2. Immunohistochemical Analysis of KORs in the NAc and VTA

**Figure 2:**
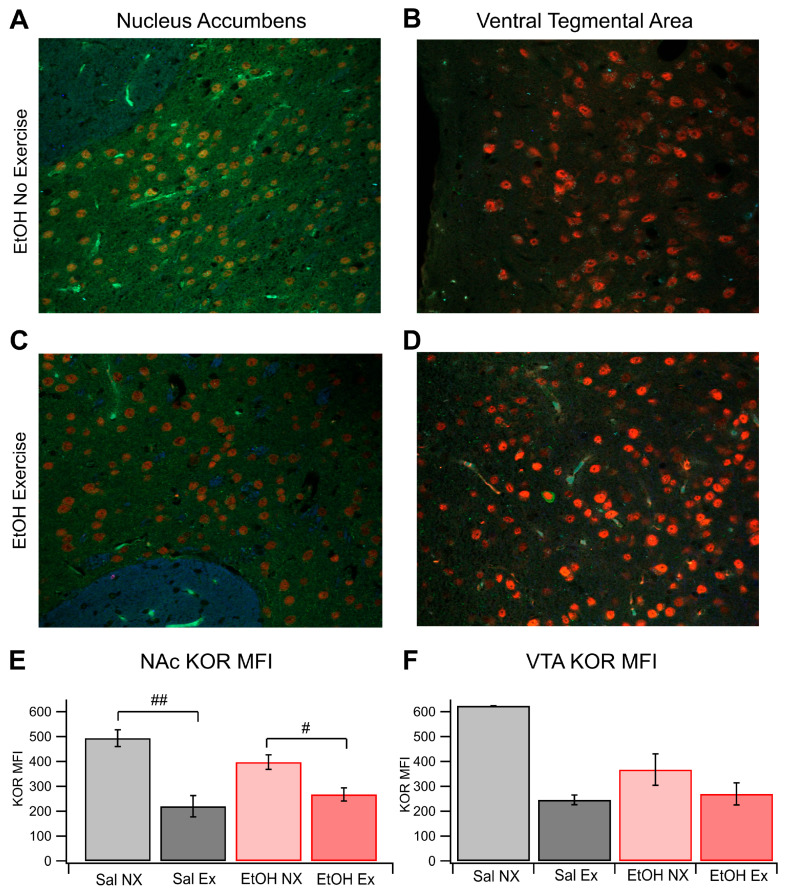
KOR FSCV. Voluntary Exercise Reduced KOR Expression in the NAc and VTA: KOR expression was evaluated immunohistochemically in four groups of mice: Sal NX, Sal EX, EtOH NX, and EtOH EX. (**A**–**D**) Example images of KOR Expression in the NAc and VTA following EtOH dependency protocol in mice with or without access to an exercise wheel for voluntary exercise (**A**–**D**). Quantification of mean fluorescence intensity of KOR fluorescence in the NAc (**E**) and VTA (**F**). Asterisks #, ## indicates *p* < 0.05, *p* < 0.01 respectively.

Brain slices were obtained from each of the four cohorts: saline no exercise (Sal NX); saline exercise (Sal EX); EtOH no exercise (EtOH NX); and EtOH exercise (EtOH EX). Brain slices containing the VTA and NAc were obtained from mice following two weeks of twice-daily 2.0 g/kg EtOH/saline injections, which we have shown previously in mice [51,52] and rats [53,54], and validated here (Figure 1), enhances EtOH consumption, an important index of EtOH dependence. Analysis revealed significant differences in the average mean fluorescent intensity (MFI) of KOR-expressing cells within the NAc across the groups (*F*_(3,18)_ = 10.93, *p* = 0.0003; Figure 2A,C,E). The average MFI of KOR-expressing cells in the EtOH no-exercise group was 398.3 ± 29.4 (*n* = 8), whereas in the EtOH with exercise group, it was 268.0 ± 26.3 (*n* = 4). In contrast, the Sal NX group showed an MFI of 494.5 ± 34.1 (*n* = 4), and the Sal EX group showed an MFI of 220.3 ± 42.9 (*n* = 6). Notably, the MFI of the EtOH EX group resembled that of the Sal EX group, both significantly differing from the EtOH and Sal NX group (6 slices analyzed per animal with multiple cells measured per slice; between EtOH NX and EtOH EX, *F*_(1,10)_ = 7.98, *p* = 0.017; between EtOH NX and Sal EX, *F*_(1,12)_ = 12.59, *p* = 0.004; between EtOH EX and Sal EX, *F*_(1,8)_ = 0.69, *p* = 0.43). Additionally, the Sal NX group exhibited a significantly increased MFI compared to the Sal EX group *F*_(1,8)_ = 20.89, *p* = 0.0018.

Expression patterns in the VTA mirrored those in the NAc but without significance (*F*_(2,12)_ = 2.00, *p* = 0.17; Figure 2B,D,F; Sal NX was excluded from this statistical test because *n* = 1 for this group). The MFI of the EtOH NX group was 368.11 ± 62.85 (*n* = 5), while the EtOH EX group showed an MFI of 270.2 ± 44.1 (*n* = 5). The Sal EX group closely aligned with the other exercise groups at 246.1 ± 19.5 (*n* = 5), whereas the Sal NX group displayed the highest MFI at 624.7 (*p* = 0.24 between EtOH NX and EtOH EX; *p* = 0.10 between EtOH NX and Sal EX; *p* = 0.63 between EtOH EX and saline EX). These findings indicate that exercise blunts the increase in expression of KORs in the NAc, but not the VTA, after chronic EtOH exposure.

### 3.3. Role of KORs in Evoked DA Release and Chronic EtOH: Fast-Scan Cyclic Voltammetry

**Figure 3:**
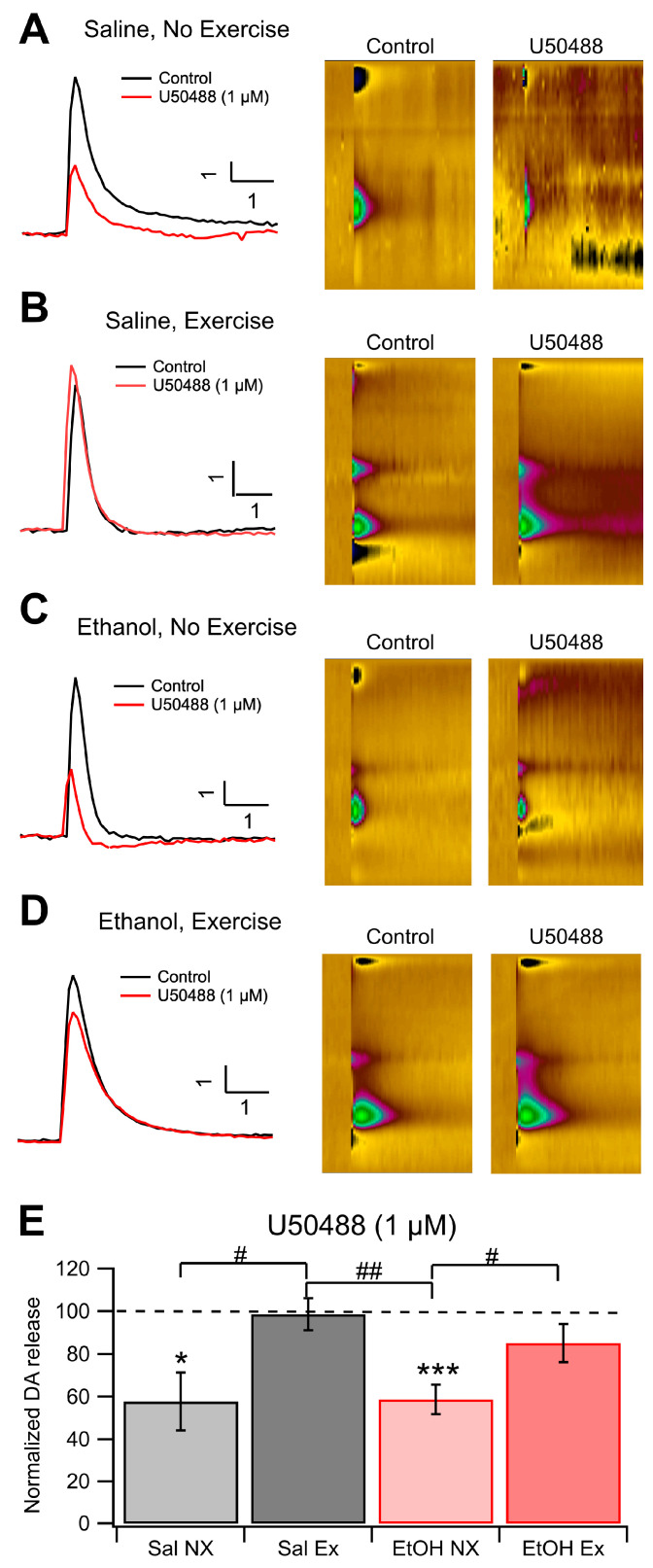
KOR IHC. Voluntary Exercise Alters KOR Sensitivity of Dopamine Release in the NAc: KOR sensitivity of DA release was evaluated with voltammetry in four groups of mice: Sal NX, Sal EX, EtOH NX, and EtOH EX. Example traces of evoked DA release following administration of 1 μM U-50488, a KOR agonist (**A**–**D**). Change in evoked DA release normalized to baseline following administration of 1 μM U50488 (**E**). Conditions indicate EtOH dependence protocol or saline protocol while being with or without access to an exercise wheel for voluntary exercise. Symbols *, ***, #, ## indicate *p* < 0.05, *p* < 0.001, *p* < 0.05, *p* < 0.01 respectively.

Dopamine release was measured using FSCV in each experimental group to determine the effects of EtOH dependence and voluntary aerobic exercise on DA dynamics. There was no change to baseline levels of DA release on average between the 4 cohorts, indicating that EtOH dependence and exercise do not affect electrically evoked levels of DA release; however, this does not give any evidence towards whether exercise affects spontaneous DA release in vivo. We measured changes in KOR sensitivity with applications of 0.3 μM and 1.0 μM U-50488, a KOR agonist, and reversal with 1.0 μM nor-BNI, a KOR antagonist. Bath administration of U-50488 significantly reduced DA release in slices taken from Sal NX mice. (*F*_(1,10)_ = 9.25, *p* = 0.012; Figure 3D,E) and also in EtOH dependent mice from the no exercise cohort (*F*_(1,19)_ = 14.90, *p* = 0.0011; Figure 3C,E). Interestingly, slices taken from mice that received aerobic exercise both EtOH dependent and naïve, did not show changes in DA release in the presence of a KOR agonist or antagonist, suggesting that exercise desensitizes KORs in the NAc (Figure 3E). Significant differences were noted between the Sal NX and Sal EX groups at the 0.3 μM and 1 μM dose level of U-50488, and between the EtOH NX and EtOH EX groups (*F*_(1,14)_ = 16.0594, *p* = 0.0013; *F*_(1,20)_ = 6.9498, *p* = 0.0158; Figure 3E). At 0.3 μM U-50488, DA release in the EtOH NX group decreased to 82.2 ± 5.6% of baseline, whereas the EtOH with exercise group decreased to 99.3 ± 5.2%. Conversely, the Sal EX group maintained evoked release at 100.6 ± 1.2%. The Sal EX group displayed reduced DA release to 69.1 ± 10.7% of the baseline. When 1 μM U-50488 was administered, the EtOH NX group dropped to 58.7 ± 6.9% of baseline and the EtOH EX group dropped to 85.1 ± 8.9%. The Sal EX group remained generally unresponsive at 98.6 ± 7.5% while the Sal NX group dropped to 57.8 ± 13.6% (*n* = 6 for EtOH NX, all others *n* = 4; *p* = 0.0263 and *p* = 0.0293 between EtOH NX and EtOH EX following bath concentrations of 0.3 μM and 1 μM U-50488, respectively; *p* = 0.031 and *p* = 0.029 between Sal NX and Sal EX following bath concentrations of 0.3 μM and 1 μM U-50488, respectively; Figure 3E). There were no significant differences between the 4 cohorts in the presence of the KOR antagonist nor-BNI (*p* > 0.05). These results suggest that exercise dampens KOR efficacy in reducing evoked DA release in both EtOH dependent and non-dependent mice.

### 3.4. Immunohistochemical Analysis of DORs in the NAc and VTA

**Figure 4:**
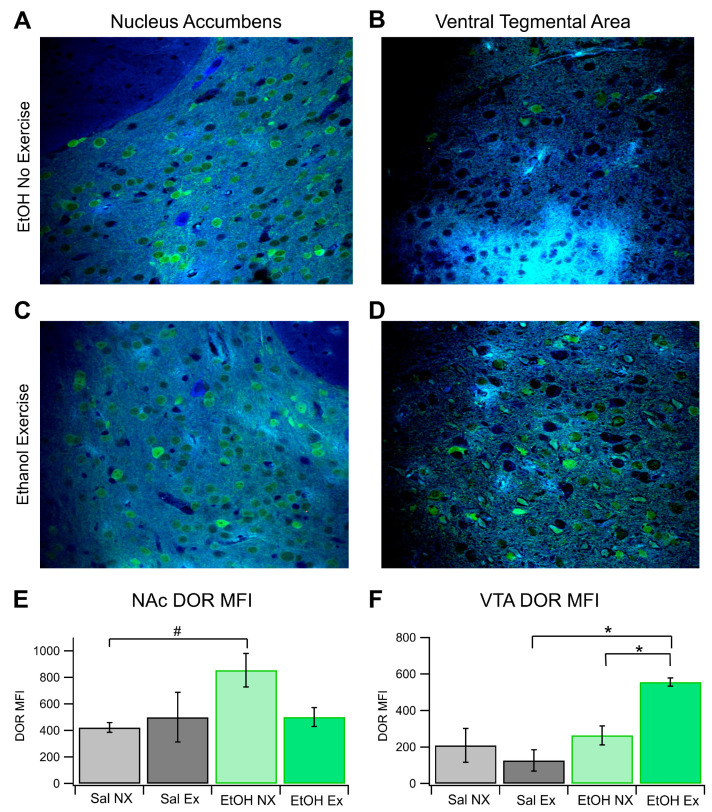
DOR FSCV. Voluntary Exercise impacted DOR Expression in the NAc and VTA: DOR expression was evaluated immunohistochemically in four groups of mice: Sal NX, Sal EX, EtOH NX, and EtOH EX. (**A**–**D**) Example images of DOR Expression in the NAc and VTA following EtOH dependency protocol in mice with or without access to an exercise wheel for voluntary exercise (**A**–**D**). Quantification of mean fluorescence intensity of DOR fluorescence in the NAc (**E**) and VTA (**F**). Asterisk *, # indicates *p* < 0.05.

Brain slices from each of the four cohorts were obtained to analyze the expression patterns of DORs in the NAc and VTA. Analysis revealed that there were no significant differences in the average MFI of DOR-expressing cells within the NAc across all groups. (*F*_(3,11)_ = 3.46, *p* = 0.054; Figure 4A,C,E). The average MFI of DOR-expressing cells in the EtOH NX group was 854.7 ± 125.9 (*n* = 4), whereas in the EtOH EX group, it was 502.1 ± 70.8 (*n* = 3). Similar to the EtOH EX group, the Sal EX group showed an MFI of 500.03 ± 186.94 (*n* = 4), and the Sal NX group showed an MFI of 422.94 ± 36.6 (*n* = 4; with 6 slices analyzed per animal with multiple cells measured per slice). Notably, there was a significant difference between the NX groups (*F*_(1,6)_ = 10.8 *p* = 0.017). The EtOH EX group (not significant, *p* = 0.07) displayed lower MFI than the EtOH NX group. This resembles the saline groups, indicating a protection or amelioration against EtOH effects. There were no significant differences in DOR expression between the EtOH EX and Sal EX groups.

Expression patterns in the VTA mirrored those in the NAc. There were significant differences between all groups in DOR MFI in the VTA (*F*_(3,9)_ = 4.98, *p* = 0.026; Figure 4B,D,F) (with 6 slices analyzed per animal with multiple cells measured per slice). The average MFI of DOR-expressing cells in the EtOH no-exercise group was 263.92 ± 51.95 (*n* = 3), whereas in the EtOH EX group, it was 556.6 ± 85.98 (*n* = 4).Similar to the EtOH NX group, the Sal NX group showed an MFI of 209.46 ± 92.75 (*n* = 4), and the Sal EX group showed an MFI of 126.39 ± 58.19 (*n* = 3). Interestingly, there was no significant difference between the NX groups in the VTA like there was in the NAc. There was, however, a significant difference between the EX groups (*F*_(1,4)_ = 10.34, *p* = 0.032). Voluntary exercise did result in a significant difference between the EtOH EX and NX groups (*F*_(1,5)_ = 7.00, *p* = 0.046). In summary, in the NAc, there was a baseline difference in DOR expression between groups that was ameliorated in the EtOH group by exercise and in the VTA we did not see the same baseline difference between chronic EtOH and naïve conditions, however exercise did change the expression of DORs in the VTA for the chronic EtOH group.

### 3.5. Role of DORs in Evoked DA Release and Chronic EtOH: Fast-Scan Cyclic Voltammetry

**Figure 5:**
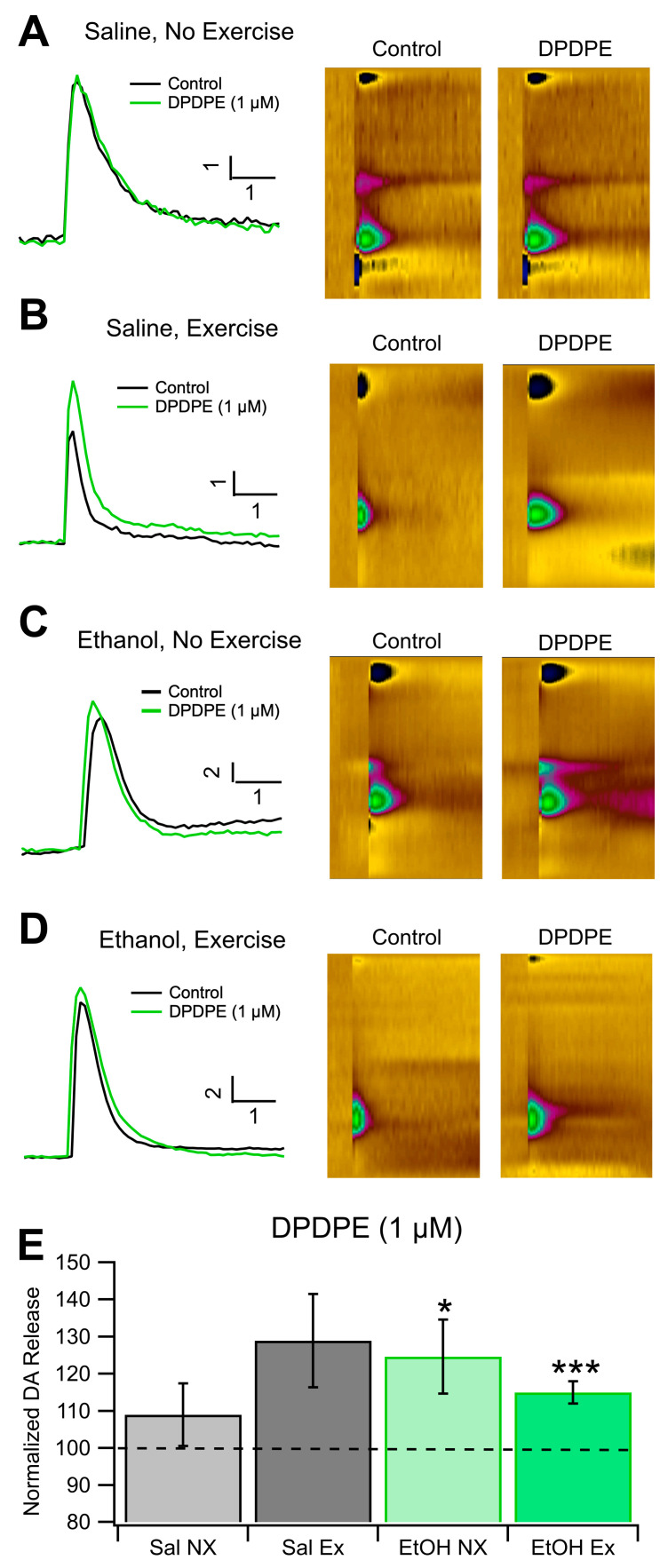
DOR IHC. Voluntary Exercise Does Not Significantly Alter DOR Sensitivity of Dopamine Release in the NAc: DOR sensitivity of DA release was evaluated with voltammetry in four groups of mice: Sal NX, Sal EX, EtOH NX, and EtOH EX. Example traces of evoked DA release following administration of 1 μM DPDPE, a DOR agonist (**A**–**D**). Change in evoked DA release normalized to baseline following administration 1 μM DPDPE, a selective DOR agonist compared between conditions (**E**) and against baseline. Conditions indicate EtOH dependence protocol or saline protocol while being with or without access to an exercise wheel for voluntary exercise. Asterisks *, *** indicates *p* < 0.05, *p* < 0.0001 respectively.

As with the analysis of KORs, we found that there were no significant differences in DA release at baseline between all groups (*n* = 4 Sal EX, Sal NX, and EtOH NX; *n* = 6 EtOH EX). This indicates that EtOH dependence and exercise do not affect electrically evoked levels of DA release, however this does not give any evidence towards whether exercise affects spontaneous DA release in vivo. We measured changes in DOR sensitivity with an application of 1.0 μM DPDPE, a DOR agonist. There were no significant differences in evoked DA release after administration of DPDE, a DOR agonist, between conditions (Figure 5E). When 1 μM DPDPE was administered, the EtOH NX group increased to 124.6 ± 9.9% of baseline and the EtOH EX group increased to 115 ± 2.98%. The Sal NX group remained generally unresponsive at 108.9 ± 8.5% while the Sal EX group increased to 128.9 ± 12.5% (*n* = 6 for EtOH EX, all others *n* = 4, Figure 5A,B,E). When compared against baseline DA release the groups dependent on EtOH (with and without exercise) did have significant increases in DA release (*F*_(1,10)_ = 25.82, *p* < 0.0001; *F*_(1,6)_ = 6.14, *p* = 0.048, respectively Figure 5C–E), but the differences between groups were not significant. The saline group did not display significant increases in DA release with the application of DPDPE. Overall, these results indicate that there were no significant changes in evoked DA release between groups receiving chronic EtOH or saline with or without exercise.

## 4. Discussion

We first demonstrated that our two week twice-daily 2.0 g/kg EtOH injection protocol was an adequate stimulus to induce EtOH dependence in mice. We have demonstrated in multiple publications that this injection protocol produces EtOH dependence in mice [51,52] and rats [53,54,55] with characteristic indices of withdrawal, as well as increased drinking. The injection procedure is more reliable than multiple weeks of chronic intermittent EtOH (CIE) vapor exposure, which we have also used to evaluate dependence using combined CIE and EtOH DID procedures in mice [51,52]. However, in this study we used the EtOH TBC procedure. The TBC drinking model allows mice to voluntary drink in moderation while the DID drinking model induces mice to voluntary consume large quantities of EtOH in a short period, which is an accepted model of binge drinking. The TBC model of moderate, prolonged drinking seemed to be a more logical approach in the context of prolonged exercise. Regardless, both of these paradigms allow for rapid screening of pharmacological agents or manipulations (e.g., exercise). In order to evaluate its utility as an index of EtOH dependence, mice were evaluated in the TBC procedure before and after twice-daily EtOH injections. Exposure to EtOH with the TBC procedure Post/Pre EtOH injections increased EtOH consumption, validating previous studies and ensuring EtOH dependence for immunohistochemical and voltametric experiments. Importantly, exercise prevented the increase in EtOH consumption using the TBC model in EtOH-dependent mice (Figure 1). Accordingly, we have shown recently that frequency-dependent mechanoreceptor activation of the cervical spine modulates VTA neuron activity and enhances DA release in the NAc via endogenous opioid effects on DORs [56,57]. We have theorized that the same specific mechanoreceptor activation is characteristic of exercise. Most importantly, using the same frequencies of cervical spine stimulation, whole-body vibration (WBV) prevents adaptations in VTA neurons, DA release, and behavioral indices associated with EtOH [55] and morphine [58] withdrawal, suggesting that specific mechanoreceptor activation, perhaps reflected in exercise, is an adequate stimulus for modulating AUD and OUD adaptations.

This study investigates the expression and sensitivity patterns of KORs and DORs and DA release in EtOH-dependent mice to elucidate the role of exercise in the natural reward pathway. Previous research has established that chronic EtOH exposure increases KOR sensitivity and upregulates the kappa/DYN system in the NAc [59,60]. Our findings for KORs are consistent with this and indicate that voluntary exercise ameliorates some of the changes that occur in mesolimbic circuity with the introduction of chronic EtOH. Our results show that although KOR sensitivity increases with chronic EtOH exposure, exercise reduces this sensitization. We also found that an increase in KOR expression in the NAc with EtOH exposure was significantly blunted by exercise. Our study reveals that voluntary exercise alone is sufficient to produce a reduction in KOR sensitivity to U-50488, a selective receptor agonist. Notably, wild mice in their natural habitat engage in regular daily exercise, suggesting that the observed reduction in sensitivity and expression may reflect a more typical state for wild mice. Conversely, the sedentary lifestyle of laboratory mice, lacking regular exercise, could contribute to alterations in KOR expression, potentially increasing susceptibility to AUD.

It is important to note that the group that only received saline and no exercise saw high KOR expression in the NAc and sensitization in their response to ex-vivo stimulation. This result was likely due to the stress of the protocol, as these mice were exposed to weekly injections without EtOH or exercise to act as a sedative or coping mechanism for them. This effect is well known among experiments that require repeated injections and self-isolation [61,62], and especially, many have found that stress of repeated injections can have its effect through the kappa/DYN system [63,64]. People with substance use disorders (SUDs) often continue their pattern of abuse partly because of altered or reduced dopamine (DA) levels, which result in feelings of dysphoria. Additionally, anxiety and stress mediated by the extended amygdala contribute to the ‘withdrawal/negative affect’ stage [65]. Anxiety/stress associated with AUD is a major risk factor for relapse [66]. The amygdala has been implicated in the anxiety/stress associated with AUD withdrawal states and constitutes allostatic dysregulation of the mesolimbic DA system (i.e., “between-system” adaptations) [67]. The recruitment of key stress neurotransmitters in the “extended amygdala” including corticotropin releasing factor (CRF) [68] and endogenous opioids like DYN results in the establishment of negative reinforcing mechanisms which play a key role in the allostatic process (i.e., “anti-reward”) [68] underlying AUD. In effect, persons with AUD drink compulsively to relieve the emotional consequences of these neurochemical dysregulations [66,67].

Consensus on DOR expression and sensitivity in the striatum is still a subject of debate and many find different results depending on the strain, length and type of EtOH exposure, and age of animal subject [69,70], As such, we found increase in DOR expression in the NAc and no changes to sensitivity with chronic EtOH, but we do not attempt to generalize these results to any other population than C57 mice in our chronic EtOH paradigm. We did see in our results, similar to that of the KORs, that the changes introduced by chronic EtOH, for example an increase in DOR expression in the NAc, was ameliorated by voluntary exercise. Interestingly, we found a strong uptick in expression of DORs in the VTA with exercise that indicate large changes to DOR expression when EtOH and exercise are combine.

We anticipated that the changes in receptor activity would result in reduced drinking behavior in mice exposed to EtOH and exercise, compared to the controls. We indeed observed a decrease in drinking among mice chronically exposed to EtOH that exercised, confirming this hypothesis. These behavioral effects indicate that these changes in sensitivity and expression translate to changes in drinking behavior after chronic EtOH exposure.

Our findings indicate that consistent aerobic exercise, promotes the translocation of DORs to cellular membranes in the VTA and perhaps a reduction of KOR sensitivity in the NAc, potentially counteracting the effects seen with chronic EtOH exposure. Mechanosensory input, mediated via the dorsal column medial lemniscus pathway, provides collateral connections outside the canonical somatosensory pathways that terminate in the somatosensory cortex. These collaterals have been shown to alter neuron firing rate in the VTA, influencing neurotransmitter release and DOR and KOR expression in the NAc [56]. In this context, we can examine the neurological implications of exercise on reward circuitry as illustrated by the results of this study.

Additional mechanistic investigations focusing on KORs and DORs in addition to MORs, and the neurological circuits responsible for the mesolimbic effects of exercise are necessary for a comprehensive understanding of exercise’s potential as an adjunctive treatment modality for AUD.

## Data Availability

The data supporting the findings of this study are available from the corresponding author upon reasonable request.

## References

[1] Koob G.F., Volkow N.D. (2010). Neurocircuitry of addiction. Neuropsychopharmacology.

[2] Wise R.A. (2008). Dopamine and reward: The anhedonia hypothesis 30 years on. Neurotox. Res..

[3] Ahn S., Phillips A.G. (2003). Independent modulation of basal and feeding-evoked dopamine efflux in the nucleus accumbens and medial prefrontal cortex by the central and basolateral amygdalar nuclei in the rat. Neuroscience.

[4] Ahn S., Phillips A.G. (2002). Modulation by central and basolateral amygdalar nuclei of dopaminergic correlates of feeding to satiety in the rat nucleus accumbens and medial prefrontal cortex. J. Neurosci..

[5] Phillips A.G., Ahn S., Howland J.G. (2003). Amygdalar control of the mesocorticolimbic dopamine system: Parallel pathways to motivated behavior. Neurosci. Biobehav. Rev..

[6] Agmo A., Federman I., Navarro V., Padua M., Velazquez G. (1993). Reward and reinforcement produced by drinking water: Role of opioids and dopamine receptor subtypes. Pharmacol. Biochem. Behav..

[7] Agmo A., Galvan A., Talamantes B. (1995). Reward and reinforcement produced by drinking sucrose: Two processes that may depend on different neurotransmitters. Pharmacol. Biochem. Behav..

[8] Wise R.A. (2004). Dopamine, learning and motivation. Nat. Rev. Neurosci..

[9] Nestler E.J. (2001). Psychogenomics: Opportunities for understanding addiction. J. Neurosci..

[10] Kalivas P.W., Churchill L., Klitenick M.A., Kalivas P.W., Barnes C.D. (1993). The circuitry mediating the translation of motivational stimuli into adaptive motor responses. Limbic Motor Circuits and Neuropsychiatry.

[11] Kalivas P.W., Volkow N.D. (2005). The neural basis of addiction: A pathology of motivation and choice. Am. J. Psychiatry.

[12] Karkhanis A., Holleran K.M., Jones S.R. (2017). Dynorphin/Kappa Opioid Receptor Signaling in Preclinical Models of Alcohol, Drug, and Food Addiction. Int. Rev. Neurobiol..

[13] Attali B., Saya D., Vogel Z. (1989). Kappa-opiate agonists inhibit adenylate cyclase and produce heterologous desensitization in rat spinal cord. J. Neurochem..

[14] Henry D.J., Grandy D.K., Lester H.A., Davidson N., Chavkin C. (1995). Kappa-opioid receptors couple to inwardly rectifying potassium channels when coexpressed by Xenopus oocytes. Mol. Pharmacol..

[15] Konkoy C.S., Childers S.R. (1989). Dynorphin-selective inhibition of adenylyl cyclase in guinea pig cerebellum membranes. Mol. Pharmacol..

[16] Prather P.L., McGinn T.M., Claude P.A., Liu-Chen L.Y., Loh H.H., Law P.Y. (1995). Properties of a kappa-opioid receptor expressed in CHO cells: Interaction with multiple G-proteins is not specific for any individual G alpha subunit and is similar to that of other opioid receptors. Brain Res. Mol. Brain Res..

[17] Tallent M., Dichter M.A., Bell G.I., Reisine T. (1994). The cloned kappa opioid receptor couples to an N-type calcium current in undifferentiated PC-12 cells. Neuroscience.

[18] Spanagel R., Herz A., Shippenberg T.S. (1992). Opposing tonically active endogenous opioid systems modulate the mesolimbic dopaminergic pathway. Proc. Natl. Acad. Sci. USA.

[19] Walker B.M., Valdez G.R., McLaughlin J.P., Bakalkin G. (2012). Targeting dynorphin/kappa opioid receptor systems to treat alcohol abuse and dependence. Alcohol.

[20] Jarman S.K., Haney A.M., Valdez G.R. (2018). Kappa opioid regulation of depressive-like behavior during acute withdrawal and protracted abstinence from ethanol. PLoS ONE.

[21] Gillett K., Harshberger E., Valdez G.R. (2013). Protracted withdrawal from ethanol and enhanced responsiveness stress: Regulation via the dynorphin/kappa opioid receptor system. Alcohol.

[22] Dakwar E., Blanco C., Lin K.H., Liu S.M., Warden D., Trivedi M., Nunes E.V. (2012). Exercise and mental illness: Results from the National Epidemiologic Survey on Alcohol and Related Conditions (NESARC). J. Clin. Psychiatry.

[23] Sirohi S., Bakalkin G., Walker B. (2012). Alcohol-induced plasticity in the dynorphin/kappa-opioid receptor system. Front. Mol. Neurosci..

[24] Anderson R.I., Lopez M.F., Griffin W.C., Haun H.L., Bloodgood D.W., Pati D., Boyt K.M., Kash T.L., Becker H.C. (2019). Dynorphin-kappa opioid receptor activity in the central amygdala modulates binge-like alcohol drinking in mice. Neuropsychopharmacology.

[25] Chefer V.I., Bäckman C.M., Gigante E.D., Shippenberg T.S. (2013). Kappa Opioid Receptors on Dopaminergic Neurons Are Necessary for Kappa-Mediated Place Aversion. Neuropsychopharmacology.

[26] Bazov I., Sarkisyan D., Kononenko O., Watanabe H., Yakovleva T., Hansson A.C., Sommer W.H., Spanagel R., Bakalkin G. (2018). Dynorphin and κ-Opioid Receptor Dysregulation in the Dopaminergic Reward System of Human Alcoholics. Mol. Neurobiol..

[27] Mitchell J.M., O’Neil J.P., Janabi M., Marks S.M., Jagust W.J., Fields H.L. (2012). Alcohol Consumption Induces Endogenous Opioid Release in the Human Orbitofrontal Cortex and Nucleus Accumbens. Sci. Transl. Med..

[28] Hirose N., Murakawa K., Takada K., Oi Y., Suzuki T., Nagase H., Cools A.R., Koshikawa N. (2005). Interactions among mu- and delta-opioid receptors, especially putative delta1- and delta2-opioid receptors, promote dopamine release in the nucleus accumbens. Neuroscience.

[29] Murakawa K., Hirose N., Takada K., Suzuki T., Nagase H., Cools A.R., Koshikawa N. (2004). Deltorphin II enhances extracellular levels of dopamine in the nucleus accumbens via opioid receptor-independent mechanisms. Eur. J. Pharmacol..

[30] Yoshida Y., Koide S., Hirose N., Takada K., Tomiyama K., Koshikawa N., Cools A.R. (1999). Fentanyl increases dopamine release in rat nucleus accumbens: Involvement of mesolimbic mu- and delta-2-opioid receptors. Neuroscience.

[31] Okutsu H., Watanabe S., Takahashi I., Aono Y., Saigusa T., Koshikawa N., Cools A.R. (2006). Endomorphin-2 and endomorphin-1 promote the extracellular amount of accumbal dopamine via nonopioid and mu-opioid receptors, respectively. Neuropsychopharmacology.

[32] Bonci A., Williams J.T. (1996). A common mechanism mediates long-term changes in synaptic transmission after chronic cocaine and morphine. Neuron.

[33] Johnson S.W., North R.A. (1992). Opioids excite dopamine neurons by hyperpolarization of local interneurons. J. Neurosci..

[34] Steffensen S.C., Stobbs S.H., Colago E.E., Lee R.S., Koob G.F., Gallegos R.A., Henriksen S.J. (2006). Contingent and non-contingent effects of heroin on mu-opioid receptor-containing ventral tegmental area GABA neurons. Exp. Neurol..

[35] Devine D.P., Wise R.A. (1994). Self-administration of morphine, DAMGO, and DPDPE into the ventral tegmental area of rats. J. Neurosci..

[36] Shippenberg T.S., Zapata A., Chefer V.I. (2007). Dynorphin and the pathophysiology of drug addiction. Pharmacol. Ther..

[37] Gunillasdotter V., Andreasson S., Hallgren M., Jirwe M. (2022). Exercise as treatment for alcohol use disorder: A qualitative study. Drug Alcohol. Rev..

[38] Droste S.K., Schweizer M.C., Ulbricht S., Reul J.M. (2006). Long-term voluntary exercise and the mouse hypothalamic-pituitary-adrenocortical axis: Impact of concurrent treatment with the antidepressant drug tianeptine. J. Neuroendocrinol..

[39] Greenwood B.N., Foley T.E., Le T.V., Strong P.V., Loughridge A.B., Day H.E., Fleshner M. (2011). Long-term voluntary wheel running is rewarding and produces plasticity in the mesolimbic reward pathway. Behav. Brain Res..

[40] Wang D., Wang Y., Wang Y., Li R., Zhou C. (2014). Impact of physical exercise on substance use disorders: A meta-analysis. PLoS ONE.

[41] Lipowski M., Szulc M., Bulinski L. (2015). Physical activity among other health-related behaviors in treatment of alcoholism. J. Sports Med. Phys. Fit..

[42] Piazza-Gardner A.K., Barry A.E. (2012). Examining physical activity levels and alcohol consumption: Are people who drink more active?. Am. J. Health Promot..

[43] Martinez D., Broft A., Foltin R.W., Slifstein M., Hwang D.R., Huang Y., Perez A., Frankle W.G., Cooper T., Kleber H.D. (2004). Cocaine dependence and d2 receptor availability in the functional subdivisions of the striatum: Relationship with cocaine-seeking behavior. Neuropsychopharmacology.

[44] Morgan D., Grant K.A., Gage H.D., Mach R.H., Kaplan J.R., Prioleau O., Nader S.H., Buchheimer N., Ehrenkaufer R.L., Nader M.A. (2002). Social dominance in monkeys: Dopamine D2 receptors and cocaine self-administration. Nat. Neurosci..

[45] Voisey J., Swagell C.D., Hughes I.P., van Daal A., Noble E.P., Lawford B.R., Young R.M., Morris C.P. (2012). A DRD2 and ANKK1 haplotype is associated with nicotine dependence. Psychiatry Res..

[46] Arida R.M., Gomes da Silva S., de Almeida A.A., Cavalheiro E.A., Zavala-Tecuapetla C., Brand S., Rocha L. (2015). Differential effects of exercise on brain opioid receptor binding and activation in rats. J. Neurochem..

[47] Vijay A., Cavallo D., Goldberg A., de Laat B., Nabulsi N., Huang Y., Krishnan-Sarin S., Morris E.D. (2018). PET imaging reveals lower kappa opioid receptor availability in alcoholics but no effect of age. Neuropsychopharmacology.

[48] Lee H., Roh S., Kim D.J. (2009). Alcohol-induced blackout. Int. J. Environ. Res. Public Health.

[49] Yorgason J.T., Espana R.A., Jones S.R. (2011). Demon voltammetry and analysis software: Analysis of cocaine-induced alterations in dopamine signaling using multiple kinetic measures. J. Neurosci. Methods.

[50] Scuppa G., Tambalo S., Pfarr S., Sommer W.H., Bifone A. (2020). Aberrant insular cortex connectivity in abstinent alcohol-dependent rats is reversed by dopamine D3 receptor blockade. Addict. Biol..

[51] Nelson A.C., Williams S.B., Pistorius S.S., Park H.J., Woodward T.J., Payne A.J., Obray J.D., Shin S.I., Mabey J.K., Steffensen S.C. (2018). Ventral Tegmental Area GABA Neurons Are Resistant to GABA(A) Receptor-Mediated Inhibition During Ethanol Withdrawal. Front. Neurosci..

[52] Williams S.B., Yorgason J.T., Nelson A.C., Lewis N., Nufer T.M., Edwards J.G., Steffensen S.C. (2018). Glutamate Transmission to Ventral Tegmental Area GABA Neurons Is Altered by Acute and Chronic Ethanol. Alcohol. Clin. Exp. Res..

[53] Gallegos R.A., Lee R.S., Criado J.R., Henriksen S.J., Steffensen S.C. (1999). Adaptive responses of gamma-aminobutyric acid neurons in the ventral tegmental area to chronic ethanol. J. Pharmacol. Exp. Ther..

[54] Ludlow K.H., Bradley K.D., Allison D.W., Taylor S.R., Yorgason J.T., Hansen D.M., Walton C.H., Sudweeks S.N., Steffensen S.C. (2009). Acute and chronic ethanol modulate dopamine D2-subtype receptor responses in ventral tegmental area GABA neurons. Alcohol. Clin. Exp. Res..

[55] Bills K.B., Otteson D.Z., Jones G.C., Brundage J.N., Baldwin E.K., Small C.A., Kim H.Y., Yorgason J.T., Blotter J.D., Steffensen S.C. (2022). Mechanical Stimulation Alters Chronic Ethanol-Induced Changes to VTA GABA Neurons, NAc DA Release and Measures of Withdrawal. Int. J. Mol. Sci..

[56] Bills K.B., Clarke T., Major G.H., Jacobson C.B., Blotter J.D., Feland J.B., Steffensen S.C. (2019). Targeted Subcutaneous Vibration With Single-Neuron Electrophysiology As a Novel Method for Understanding the Central Effects of Peripheral Vibrational Therapy in a Rodent Model. Dose Response.

[57] Bills K.B., Obray J.D., Clarke T., Parsons M., Brundage J., Yang C.H., Kim H.Y., Yorgason J.T., Blotter J.D., Steffensen S.C. (2020). Mechanical stimulation of cervical vertebrae modulates the discharge activity of ventral tegmental area neurons and dopamine release in the nucleus accumbens. Brain Stimul..

[58] Jones G.C., Small C.A., Otteson D.Z., Hafen C.W., Breinholt J.T., Flora P.D., Burris M.D., Sant D.W., Ruchti T.R., Yorgason J.T. (2023). Whole-Body Vibration Prevents Neuronal, Neurochemical, and Behavioral Effects of Morphine Withdrawal in a Rat Model. Int. J. Mol. Sci..

[59] Anderson R.I., Lopez M.F., Becker H.C. (2016). Stress-Induced Enhancement of Ethanol Intake in C57BL/6J Mice with a History of Chronic Ethanol Exposure: Involvement of Kappa Opioid Receptors. Front. Cell. Neurosci..

[60] Melchior J.R., Jones S.R. (2017). Chronic ethanol exposure increases inhibition of optically targeted phasic dopamine release in the nucleus accumbens core and medial shell ex vivo. Mol. Cell Neurosci..

[61] Du Preez A., Law T., Onorato D., Lim Y.M., Eiben P., Musaelyan K., Egeland M., Hye A., Zunszain P.A., Thuret S. (2020). The type of stress matters: Repeated injection and permanent social isolation stress in male mice have a differential effect on anxiety- and depressive-like behaviours, and associated biological alterations. Transl. Psychiatry.

[62] Drude S., Geißler A., Olfe J., Starke A., Domanska G., Schuett C., Kiank-Nussbaum C. (2011). Side effects of control treatment can conceal experimental data when studying stress responses to injection and psychological stress in mice. Lab. Anim..

[63] Flaisher-Grinberg S., Persaud S.D., Loh H.H., Wei L.-N. (2012). Stress-induced epigenetic regulation of κ-opioid receptor gene involves transcription factor c-Myc. Proc. Natl. Acad. Sci. USA.

[64] Shirayama Y., Ishida H., Iwata M., Hazama G.I., Kawahara R., Duman R.S. (2004). Stress increases dynorphin immunoreactivity in limbic brain regions and dynorphin antagonism produces antidepressant-like effects. J. Neurochem..

[65] Koob G.F. (2003). Alcoholism: Allostasis and beyond. Alcohol. Clin. Exp. Res..

[66] Koob G.F. (2009). Neurobiological substrates for the dark side of compulsivity in addiction. Neuropharmacology.

[67] Koob G.F. (2009). Brain stress systems in the amygdala and addiction. Brain Res..

[68] Koob G.F. (2010). The role of CRF and CRF-related peptides in the dark side of addiction. Brain Res..

[69] Hynes M.D., Lochner M.A., Bemis K.G., Hymson D.L. (1983). Chronic ethanol alters the receptor binding characteristics of the delta opioid receptor ligand, D-Ala2-D-Leu5 enkephalin in mouse brain. Life Sci..

[70] Alongkronrusmee D., Chiang T., Van Rijn R.M. (2016). Delta Opioid Pharmacology in Relation to Alcohol Behaviors.

[71] Brundage J.N. (2020). Aerobic Exercise Alters Opioid Receptors Following Chronic Alcohol Exposure. Master’s Thesis.

